# The association of high-normal international-normalized-ratio (INR) with mortality in patients referred for coronary angiography

**DOI:** 10.1371/journal.pone.0221112

**Published:** 2019-08-15

**Authors:** Graciela E. Delgado, Andreas Zirlik, Rudolf Gruber, Thomas Scheffold, Bernhard K. Krämer, Winfried März, Marcus E. Kleber

**Affiliations:** 1 Vth Department of Medicine, Medical Faculty Mannheim, Heidelberg University, Mannheim, Germany; 2 University Heart Centre Freiburg University, Department of Cardiology and Angiology I, Faculty of Medicine, University of Freiburg, Freiburg, Germany; 3 Hospital of the Order of St.John of God, Central Laboratory, Regensburg, Germany; 4 MediClin Medical Care Centre, Department of Cardiology, Lahr, Germany; 5 Clinical Institute of Medical and Chemical Laboratory Diagnostics, Medical University Graz, Graz, Austria; 6 Synlab Academy, Synlab Holding Deutschland GmbH, Mannheim, Germany; Beijing Key Laboratory of Diabetes Prevention and Research, CHINA

## Abstract

**Aims:**

The international-normalized-ratio (INR) is typically used to monitor patients on warfarin or related oral anticoagulant therapy. The aim of our study was to investigate the association of the INR with mortality in coronary artery disease (CAD) patients not on oral anticoagulant therapy.

**Methods and results:**

Between 1997 to 2000 the LUdwigshafen RIsk and Cardiovascular Health (LURIC) study enrolled 3316 patients of German ancestry that had been referred for coronary angiography. We excluded patients on coumarin therapy (n = 222) and patients with an INR more than 5 standard deviations (SD) away from the mean (n = 30). During a median follow-up of 9.9 years, 884 patients died, 547 patients from cardiovascular causes. After adjustment for cardiovascular risk factors the INR was associated with all-cause mortality in all patients and the CAD positive group with HRs (95% CI) of 1.14(1.07–1.21) and 1.16(1.09–1.23) per 1-SD increase, respectively. Adjustment for NT-proBNP rendered the association insignificant.

**Conclusion:**

In LURIC, the INR was positively associated with mortality in patients with prevalent CAD not on oral anticoagulant therapy as well as in patients without CAD. Adjustment for NT-proBNP abolished the association suggesting clinical or subclinical heart failure strongly contributing to increased INR and higher mortality.

## Introduction

In 1935 Dr Armand Quick and colleagues developed the prothrombin time, the time (in seconds) it takes plasma to clot after addition of tissue factor (also known as Quick test) [1]. Tom Kirkwood determined in the early 1980s the international normalized ratio (INR) to provide a more consistent way of measuring the function of the coagulation system [2]. Each prothrombin reagent is assigned an ISI value (International Sensitivity Index) by the manufacturer, which indicates how a reagent compares to an international reference tissue factor. The INR became widely accepted after its approval by the World Health Organization. It is typically used for monitoring patients on warfarin or related oral anticoagulant therapy. The normal range for a healthy person not using vitamin K antagonists is 0.8–1.2, while on oral anticoagulant therapy the accepted target usually is in the range of 2.0–3.0. A high INR indicates a higher risk of bleeding, while a lower INR suggests a higher risk of thrombosis. In patients receiving oral anticoagulant therapy, e.g. patients suffering from atrial fibrillation and/or increased stroke risk, the INR is a useful predictor not only for bleeding but also for mortality [3, 4]. In patients with acutely decompensated heart failure, it has been shown to be an independent predictor of all-cause mortality [5]. Recently, the association of INR on admission with the 90-day mortality of critically ill patients who underwent endarterectomy during hospitalization has been shown [6], and also that the prothrombin time-albumin ratio (PTAR) predicted 90-day mortality in critically ill patients with liver cirrhosis [7]. Another study reported that prolonged initial prothrombin time or higher INR in the absence of anticoagulant therapy was associated with all-cause mortality in patients with acute coronary syndrome who were undergoing percutaneous coronary intervention [8] and the INR has been shown to be associated with mortality in acute pulmonary embolism [9].

The INR is an essential component of the Model for end-stage liver disease (MELD) score that is used to estimate the severity of liver disease and to predict mortality in end-stage liver disease patients [10]. However, to the best of our knowledge the utility of the INR as predictor of mortality has not been investigated in patients with coronary artery disease (CAD) including patients with stable CAD who did not receive oral anticoagulation. In these patients, a lower thrombogenic potential would usually be regarded as beneficial. Therefore, it was the aim of our study to investigate the association of the INR with all-cause and cardiovascular mortality in patients with and without CAD that participated in the Ludwigshafen Risk and Cardiovascular Health (LURIC) study. We found an increased mortality risk with increasing INR.

## Materials and methods

### Study population

The LUdwigshafen RIsk and Cardiovascular Health (LURIC) study is an ongoing prospective study of 3316 patients of German ancestry who had an indication for coronary angiography. Patients were recruited between 1997 and 2000 at the Ludwigshafen Cardiac Centre [11]. All patients were clinically stable (except for acute coronary syndromes). Inclusion criteria were: German ancestry, clinical stability except for acute coronary syndromes and the availability of a coronary angiogram. Exclusion criteria were: any acute illness other than acute coronary syndromes, any chronic disease where non-cardiac disease predominated and a history of malignancies within the past five years.

Patients receiving coumarins (n = 222, 6.7%) and patients with an INR more than 5 standard deviations (SD) away from the mean (n = 30) were excluded from the analyses leading to a final sample size of 3064. **Figure A in S1 File** shows the INR distribution in LURIC. The investigation conforms to the principles outlined in the *Declaration of Helsinki* (Br Med J 1964; **ii:** 177). The LURIC study was approved by the ‘Landesärztekammer’ Ethics Committee of the Rheinland-Pfalz state in Germany. All patients signed informed written consent at study entry.

### Laboratory procedures

Fasting blood samples were taken by venipuncture in the early morning prior to angiography. Aliquots were frozen at -80°C. The Prothrombin time (quick) was determined using a clotting assay (calcium thromboplastin for total activity of FII, V, VII and X STA Neoplastin Plus/STA, Stago Diagnostics/Roche, Mannheim, Germany). The aPTT was determined using the STA APTT Kaolin assay on a STA Stago analyser (Stago Diagnostics/Roche, Mannheim, Germany). Endogenous thrombin potential (ETP) was determined using INNOVANCE ETP on a BCS coagulation analyser (Siemens Healthcare Diagnostics Inc., Germany). Factors II, V, VII, VIII, IX, XI, XII and XIII were measured using plasma deficient for the respective factor on a STA Stago analyser (Immuno GmbH, Heidelberg, Germany). Fibrinogen was determined using the STA fibrinogen assay on a STA Stago analyser (Stago Diagnostica/Roche Mannheim, Germany). Plasmin activator inhibitor (PAI-1) activity, tissue plasmin activator (tPA) activity and tPA/PAI-1 complexes were measured using the Chromolize PAI-1, Chromolize tPA and TintElize tPA-PAI-1 assays on a SLT Spectra TECAN analyser (Biopool, Umea, Sweden). Tissue factor pathway inhibitor (TFPI) was measured using the Actichrome TFPI Activity assay (American Diagnostica, Inc., Greenwich, USA). Von Willebrand factor (vWF) was determined using the STA Liatest vWF (Stago Diagnostica/Roche, Mannheim, Germany). Protein C was determined using the COAMATIC assay on a STA Stago analyser (Chromogenix Instrumentation, Laboratory SpA, Milan, Italy). D-Dimers were determined using the STA LIATEST D-DI assay (Roche, Mannheim, Germany). Antithrombin 3 was determined using the STA antithrombin III assay (Stago Diagnostica/Roche, Mannheim, Germany).

GOT, total bilirubin and cholinesterase were measured on a Hitachi 717 analyser using the AST (ASAT/GOT), BIL-T and CHE assay, respectively.

High sensitive C-reactive protein was determined using the N LATEX CRP mono assay on a Behring nephelometer II (Dade Behring GmbH Marburg, Germany). NT-pro-BNP was measured by electro-chemoluminescence on an Elecsys 2010 (Roche Diagnostics). ICAM-1 and VCAM-1 were determined using Human soluble ICAM-1 and Human soluble VCAM-1 assays (R&D Systems GmbH, Wiesbaden, Germany). Free thyroxine (fT4) and free triiodothyroxine (fT3) were determined on an Elecsys 2010 Roche autosampler (Roche Mannheim, Germany).

### Clinical definitions and endpoints

The presence of a visible luminal narrowing (>20% stenosis) detected by angiography in at least one of 15 coronary segments according to the classification of the American Heart Association was used to define coronary artery disease (CAD). Type 2 diabetes mellitus was defined according to 2010 guidelines of the American Diabetes Association as increased fasting (≥126 mg/dl) and/or post-challenge (2 h after the 75 g glucose load ≥200 mg/dl) glucose and/or elevated glycated haemoglobin (>6.5%) and/or a history of diabetes. Hypertension was defined as a systolic and/or diastolic blood pressure ≥140 and/or ≥90 mm Hg or a significant history of hypertension. The glomerular filtration rate was estimated by using the 2012 CKD-EPI eGFRcreat-cys equation. The fatty liver index was calculated as described by Bedogni et al. [12].

### Follow-up

Information on vital status was obtained from local registries. Death certificates were obtained in 97% of deceased participants. Two experienced clinicians who were blinded to patient characteristics and who classified the causes of death reviewed death certificates, medical records of local hospitals and autopsy data independently. In cases of disagreement or uncertainty concerning the coding of a specific cause of death the decision was made by a principal investigator (W.M.). During a median follow-up of 9.9 years (8.8–10.7), 884 patients (28.9%) died, 547 patients (17.9%) from cardiovascular causes. Cardiovascular mortality (CVM) included the following categories: sudden cardiac death (SCD; n = 223, 7.3%), fatal myocardial infarction (n = 98, 3.2%), death due to congestive heart failure (n = 128, 4.2%), death after intervention to treat coronary artery disease (n = 23, 0.8%), fatal stroke (n = 54, 1.8%), and other causes of death due to CAD (n = 18, 0.6%). Information for vital status is complete for all participants but the cause of death of 20 deceased persons was unknown and these patients were included in calculations of all-cause mortality, but not in calculations considering different causes of death.

### Statistical analyses

All continuous variables were checked for normality by visual inspection of the distribution and comparing mean and median values. Variables showing a skewed distribution were logarithmically transformed before entering analyses. Continuous variables were compared between groups by ANOVA. Associations between categorical variables were examined by chi-square testing. The INR was examined as tertiles or as standardized, Z-transformed values. To examine the relationship with mortality Kaplan-Meier curves were produced to evaluate the cumulative survival during follow-up, according to tertiles of INR.

Hazard ratios and 95% confidence intervals (95% CI) were calculated using the Cox proportional hazards model. Multivariable adjustment was carried out as indicated. The proportional hazard assumption was checked by examination of scaled Schoenfeld residuals. Results are shown per 1 standard deviation increase in INR or per tertile, using the first tertile as reference. Hazard ratio plots were drawn using the R-package ‘rms’ (v5.1–1) with INR modelled as restricted cubic spline with three knots. Automated model selection was performed using the R package ‘bootstepAIC’ (v1.2–0) using 100 bootstrap runs. All variables had been Z-transformed before analysis to get estimates per 1-SD increase of each variable. ROC curves were calculated and compared using the method of Delong as implemented in the R package ‘pROC’ (v1.8). Two-sided P-values < 0.05 were used to indicate statistical significance. IBM SPSS Statistics v. 22.0 (IBM Corporation) and R statistical software v. 3.4.0 (http://www.r-project.org) was used for all analyses

## Results

### Association of the INR with clinical and biochemical markers

Participants with higher INR at baseline were older and more often male than participants with a lower INR (**Table 1**). Higher INR was associated with lower LDL-C, HDL-C, blood pressure and eGFR, whereas high-sensitivity C-reactive protein (hsCRP) and N-terminal pro-brain-natriuretic peptide (NT-proBNP) were higher. Regarding markers of liver function, higher INR was associated with higher GOT, total bilirubin and MELD-XI score but lower albumin, cholinesterase and fatty liver index. We also examined a number of proteins linked to the coagulation system and found a direct association of the INR with FVIII, von Willebrand factor, tPA and D-dimers, whereas there was an inverse association with FII (prothrombin), FV, FVII and PAI-1. The percentage of participants suffering from diabetes mellitus, coronary artery disease or heart failure increased in higher INR tertiles. Medication use in the study participants is shown in **Table A in S1 File**.

**Table 1:** Study characteristics according to tertiles of INR (mean (SD) or median (25^th^-75^th^ percentile).

	International normalized ratio			
	1st tertile	2nd tertile	3rd tertile	
Variable	(0.70–1.02)	(1.02–1.08)	(1.08–1.79)	P
Age (years)	60.7(10.2)	62.9(10.8)	64.1(10.9)	<0.001
Male sex (%)	64.5	69.1	76.5	<0.001
BMI (kg/m^2^)	27.7(4.16)	27.5(3.94)	27.2(4.09)	0.018
LDL-C (mg/dl)	120(36.1)	117(32.5)	113(33.2)	<0.001
HDL-C (mg/dl)	40.4(11.2)	39(10.7)	36.5(10)	<0.001
TG (mg/dl)	146(109–202)	148(111–201)	146(109–202)	0.808
systolic BP (mmHg)	141(22.6)	143(23.8)	140(24.5)	0.002
diastolic BP (mmHg)	81.6(11.2)	81.4(11.4)	79.9(11.8)	0.004
Fasting glucose (mg/dl)	103(95–118)	102(93–118)	102(93–119)	0.739
hsCRP (mg/l)	3.08(1.3–7.31)	3.32(1.26–8.35)	3.87(1.32–10.3)	<0.001
NT-proBNP (ng/ml)	187(83–473)	273(99–724)	476(157–1490)	<0.001
Albumin (g/dl)	4.46(0.54)	4.39(0.55)	4.28(0.56)	<0.001
Cholinesterase (U/l)	6011(1274)	5786(1253)	5291(1342)	<0.001
GOT (U/l)	11.3(6.53)	11.7(7.12)	12.4(9.54)	0.023
Bilirubin (mg/dl)	0.56(0.3)	0.62(0.31)	0.73(0.49)	<0.001
MELD-XI	4.8(2.1–7.0)	5.6(3.3–8.0)	6.4(4.2–9.0)	<0.001
Fatty Liver Index	54.8(27.2)	52.6(26.2)	51.2(27.4)	0.011
FII (U/dl)	115(18.7)	110(17.4)	99.4(21.1)	<0.001
FV (U/dl)	125(19.6)	115(19.2)	103(19.5)	<0.001
FVII (U/dl)	137(25.4)	126(22.2)	109(23.7)	<0.001
FVIII (U/dl)	171(66.9)	175(69.1)	182(72.2)	0.002
von Willebrand (U/dl)	158(66.4)	165(68.6)	181(79.7)	<0.001
tPA/PAI-1 complex (μg/l)	8.25(3.99)	7.82(3.80)	7.94(4.04)	0.055
tPA activity (U/l)	0.59(0.30–0.92)	0.63(0.35–1.02)	0.70(0.39–1.15)	<0.001
PAI-1 activity (U/ml)	21(12–37.5)	18(10–34)	15(8–30)	<0.001
D-Dimer (mg/l)	0.34(0.22–0.57)	0.36(0.22–0.63)	0.39(0.22–0.76)	0.002
Fibrinogen (mg/dl)	393(95.3)	397(108)	399(121)	0.421
aPTT (sec)	32(30–34)	33(31–35)	35(32–39)	<0.001
ETP (nmol*min)	105(18.9)	101(21.8)	89.3(29.1)	<0.001
Platelets (/nl)	241(62.9)	233(65.2)	226(75.8)	<0.001
eGFR (ml/min/1.73 m^2^)	84.8(19.2)	82.7(19.8)	78.9(20.6)	<0.001
Diabetes mellitus (%)	35.6	41.4	41.9	0.005
Coronary artery disease (%)	75.1	80.9	80.1	0.002
Heart failure (%)	23.2	27.4	41.4	<0.001
reduced/preserved EF (%)	12.7 / 10.5	14.1 / 13.3	24.1 / 17.3	
Hypertension (%)	73	73.3	72.5	0.919
Venous thrombosis / pulmonary embolism (%)	93.8	95.6	94.4	0.152
Smoking (active/ex/never, %)	28.2/38.9/32.9	23.9/39.5/36.6	19.7/43.9/36.4	<0.001
Prothrombin variant (homozygous/heterozygous, %)	0/3.8	0/3.2	0.1/2.8	0.402
Factor V Leiden (homozygous/heterozygous, %)	0.2/9.3	0.1/6.9	0.2/7	0.191

*ANOVA for continuous variables (non-normally distributed variables were log transformed before entering analyses), χ^2^-test for categorical variables.

### Independent predictors of INR

To examine in more detail which variables had the strongest impact on the INR we created a linear regression model including a large number of cardiovascular risk factors, markers of liver function and coagulation factors. The following variables were retained in the final model: Age, sex, HDL-C, FII, FV, FVII, FVIII, FXIII, AT3, protC, protS, TFPI, hsCRP, albumin, GOT, MELD-XI, platelet count, ICAM-1, VCAM-1, free T3 (**Table 2**). The strongest impact on INR had the factors VII, V and II.

**Table 2:** Estimates of a linear regression model of INR.

Variable	Estimate	Std.Error	t	P-value
FVII	-0.037152	0.001613	-23.034	<0.001
FV	-0.026471	0.001747	-15.148	<0.001
FII	-0.022298	0.002215	-10.067	<0.001
hsCRP	0.01014	0.001462	6.934	<0.001
Platelets	0.008628	0.00139	6.208	<0.001
ProtC	0.013186	0.002221	5.937	<0.001
FXIII	0.007896	0.001384	5.706	<0.001
Age	0.00788	0.001472	5.352	<0.001
MELD-XI	0.00817	0.001528	5.347	<0.001
VCAM-1	0.010545	0.002176	4.846	<0.001
free T3	0.006063	0.001323	4.584	<0.001
GOT	0.00601	0.001461	4.113	<0.001
TFPI	0.005134	0.001365	3.762	<0.001
Sex	0.01243	0.003382	3.675	<0.001
AT3	-0.005145	0.001515	-3.397	0.001
HDL-C	-0.004669	0.001408	-3.315	<0.001
free ProtS	0.005884	0.001792	3.283	0.001
FVIII	0.004349	0.00149	2.919	0.004
ICAM-1	-0.005561	0.002017	-2.757	0.006
Albumin	-0.003692	0.001397	-2.644	0.008

### Association with mortality

#### All-cause and cardiovascular mortality per 1-SD increase

**Fig 1:**
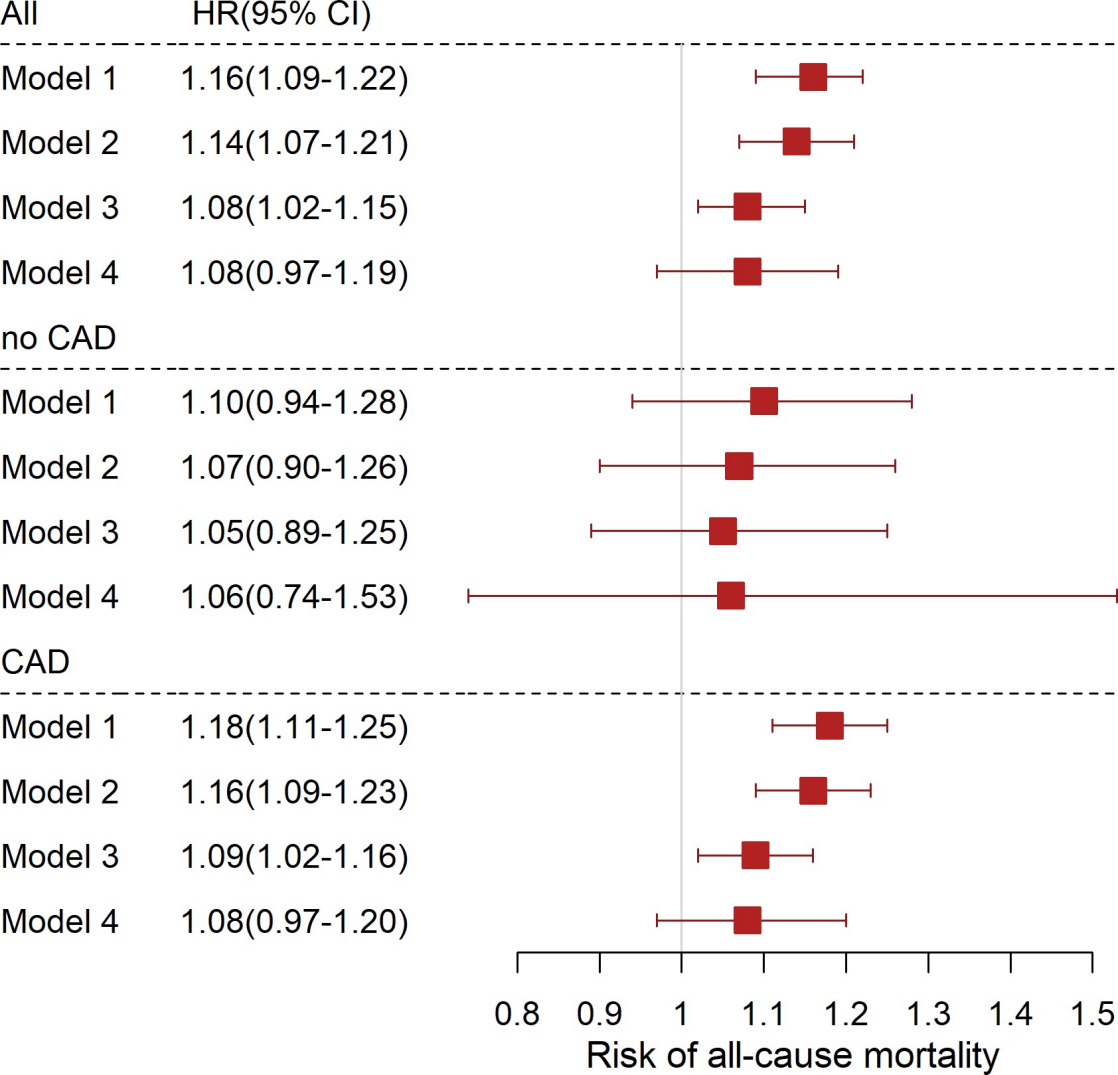
Association of INR and all-cause mortality. Cox proportional hazard regression has been used to calculate the risk of all-cause mortality per 1-SD increase in the INR. Model 1: adjusted for age and gender; model 2: additionally adjusted for BMI, LDL-C, HDL-C, hypertension, diabetes mellitus, smoking status and the use of antiplatelet and lipid lowering drugs; model 3: additionally adjusted for MELD-XI score and history of CAD or heart failure; model 4: model 3 + NT-proBNP.

During a median follow-up of 9.9 years (8.8–10.7), 884 (28.9%) LURIC participants died, 547 (17.9%) from cardiovascular causes. To examine the association of the INR with mortality we calculated hazard ratios (95% confidence intervals) per 1-SD increase in INR using the Cox proportional hazards model adjusted for age and gender (model 1) or additionally adjusted for BMI, LDL-C, HDL-C, diabetes mellitus, hypertension, smoking and the use of antiplatelet or lipid lowering drugs (model 2). After adjustment for cardiovascular risk factors the INR was positively associated with all-cause mortality in all patients and the CAD positive group with HRs (95% CI) of 1.14(1.07–1.21) and 1.16(1.09–1.23) per 1-SD increase, respectively (**Fig 1**). In persons without angiographic CAD, the INR was also positively associated with all-cause mortality, but this did not reach statistical significance, possibly due to a smaller number of events occurring in this subgroup. As a higher INR has been reported in patients with severe heart failure or liver disease we additionally adjusted for the Model for end-stage liver disease excluding INR (MELD-XI) score [13] as well as prevalent heart failure and coronary artery disease (model 3). The association of INR with mortality was attenuated but remained significant. In a further step, we also adjusted for NT-proBNP as a marker of heart failure (model 4) which further attenuated the association.

Cardiovascular mortality increased in parallel to higher INR with HRs of 1.23(1.01–1.51) and 1.21(1.13–1.30) per 1-SD increase in CAD positive and CAD negative patients, respectively (**Figure B in S1 File**). We also stratified for the presence of heart failure or acute coronary syndrome (**Figures C and D in S1 File**). Trends were similar for healthy and diseased study participants with the healthy participants showing a slightly stronger association.

#### Hazard ratio plots

**Fig 2:**
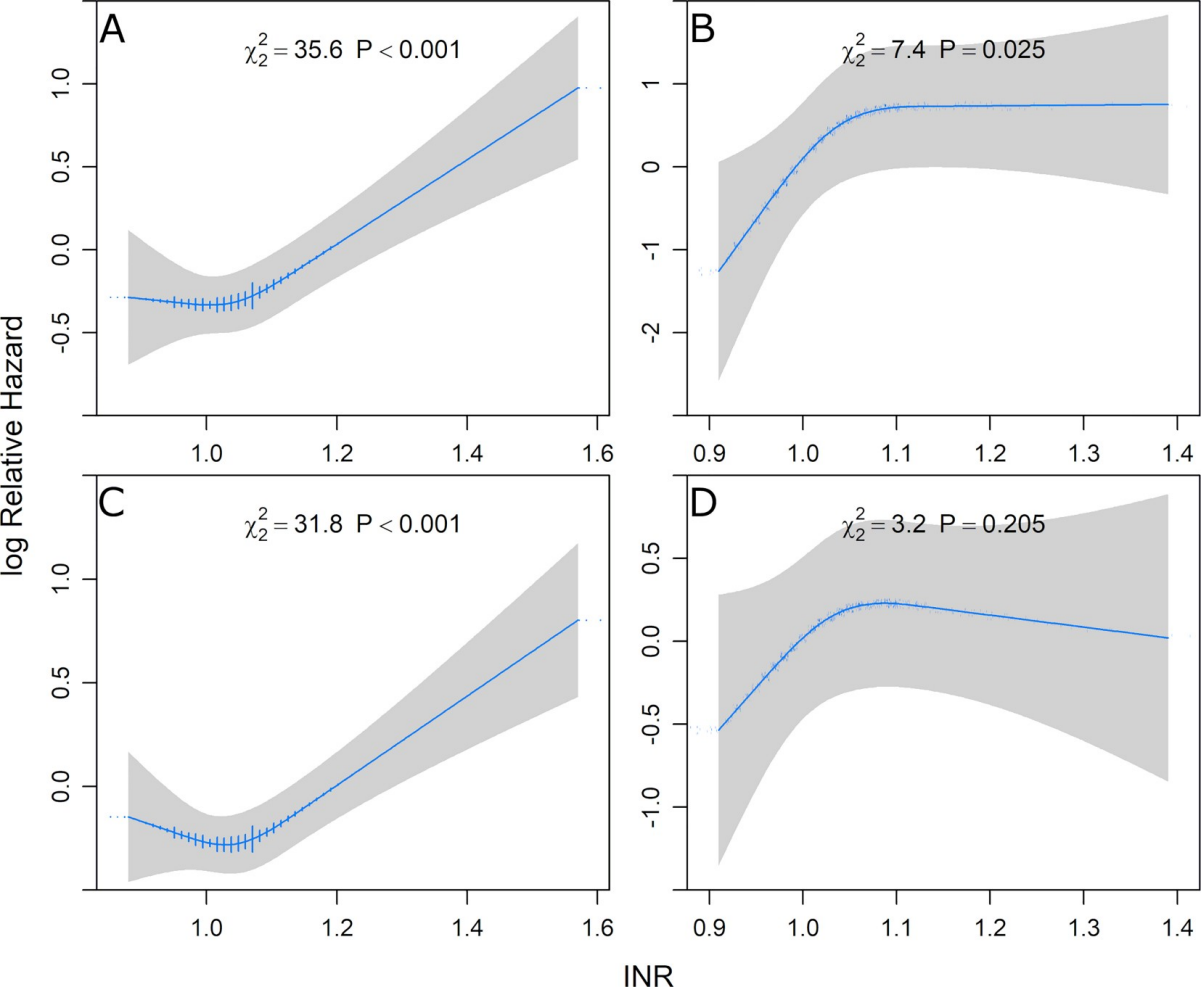
Relationship between INR and mortality. Cox proportional hazards regression including the INR modelled as restricted cubic splines with three knots with adjustment for age, gender, BMI, LDL-C, HDL-C, diabetes mellitus, hypertension, smoking status and the use of antiplatelet and lipid lowering drugs has been used to analyze the association of the INR with cardiovascular mortality (A+B) and all-cause mortality (C+D), separately for CAD positive and CAD negative patients. The log relative hazard is plotted against the INR value.

To analyse the relationship between the INR and mortality in more detail we generated hazard ratio plots modelling the INR as restricted cubic spline with three knots (**Fig 2**). In the CAD patients there appears to be a slightly J-shaped curve with the lowest risk at an INR of approximately 1.05 and linearly increasing risk at higher values. In the CAD negative patients the risk increases only up to an INR of about 1.1 but confidence intervals broaden with higher INR values. The INR was the fourth most important variable in the multiple adjusted model 3 following age, diabetes mellitus and smoking in CAD patients while it ranked only sixth in patients free of CAD at baseline (**Figure E in S1 File**).

#### Stratification into tertiles

**Fig 3:**
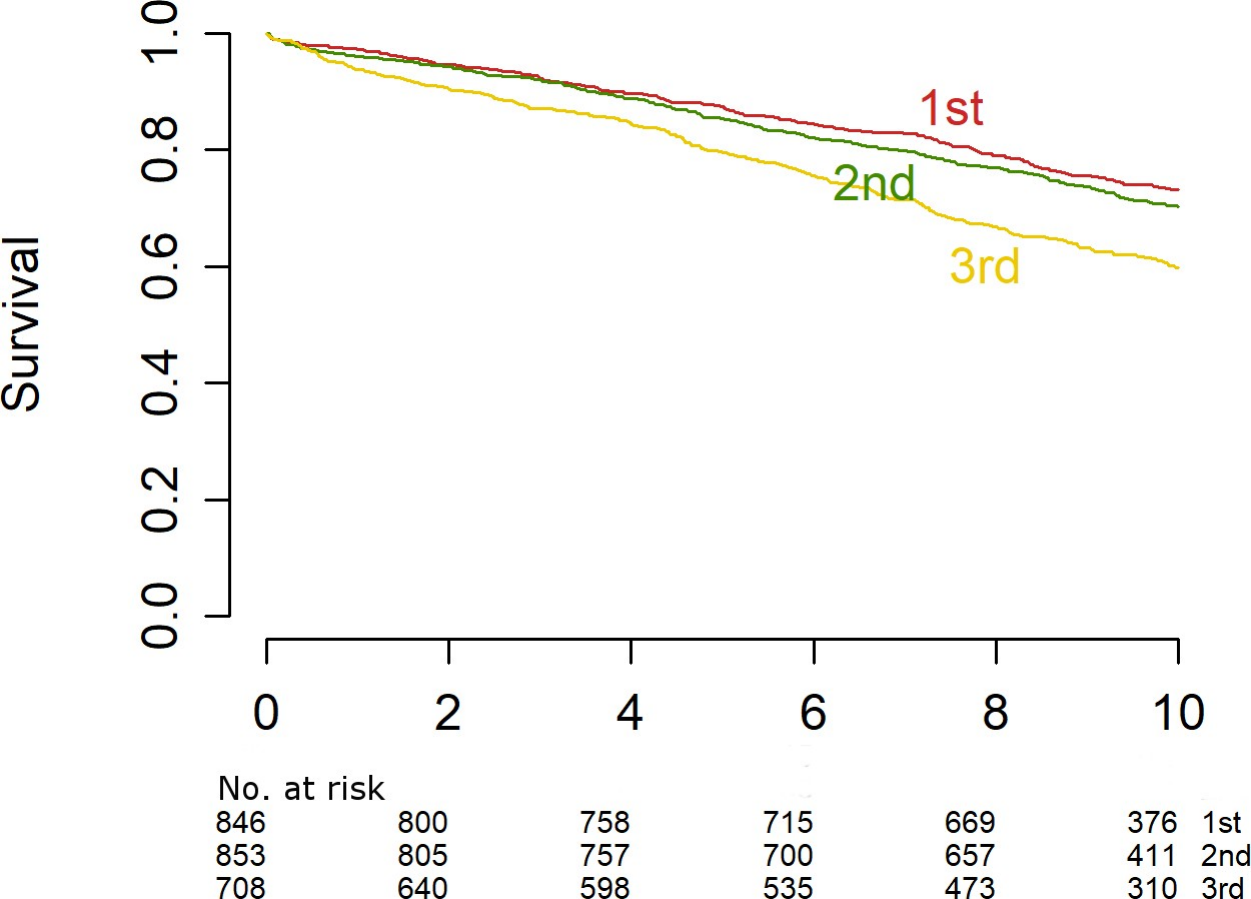
Survival curves showing the association of INR tertiles with all-cause mortality in LURIC patients with coronary artery disease. The first tertile (0.7–1.02) is drawn as a red line, the second tertile (1.02–1.08) as a green line and the third tertile (1.08–1.79) as a yellow line. The table below shows the number of study participants at risk to the respective time points.

Because of the nonlinear relationship between INR and mortality, we calculated tertiles of INR in the whole cohort as well as subgroup specific tertiles for CAD positive and CAD negative patients. Kaplan-Meier curves show the association of the highest INR tertile with reduced survival in CAD patients (**Fig 3**).

In Cox regression analysis adjusted for cardiovascular risk factors the highest INR tertile was consistently associated with increased risk in all groups with HRs of 1.26 (1.07–1.50), 1.24 (1.03–1.48) and 1.51 (0.92–2.49) for all patients, CAD positive and CAD negative patients, respectively (**Table 3**). We also used the thromboplastin time instead of the INR and also analysed the association of a second global coagulation test, the activated partial thromboplastin time (aPTT), with mortality and cardiovascular mortality (**Tables B and C in S1 File**). We observed similar tendencies towards increased risk with thromboplastin time and higher aPTT in CAD patients whereas in patients free of CAD the second aPTT tertile was associated with the highest risk. Patient characteristics according to heart failure status are shown in **Table D in S1 File**.

**Table 3:** All-cause mortality and cardiovascular mortality according to tertiles of INR.

		All-cause mortality		Cardiovascular mortality	
		HR (95% CI)	*p*	HR (95% CI)	*P*
***All participants (n = 3064)***					
Model 1	1^st^ (≤1.01)	1^reference^		1^reference^	
	2^nd^ (1.02–1.08)	1.02 (0.86–1.21)	0.800	1.04 (0.83–1.29)	0.746
	3^rd^ (≥1.09)	1.34 (1.14–1.58)	<0.001	1.56 (1.26–1.92)	<0.001
Model 2	1^st^ (≤1.01)	1^reference^		1^reference^	
	2^nd^ (1.02–1.08)	0.96 (0.81–1.14)	0.678	0.97 (0.77–1.20)	0.758
	3^rd^ (≥1.09)	1.26 (1.07–1.50)	0.006	1.45 (1.17–1.80)	<0.001
Model 3	1^st^ (≤1.01)	1^reference^		1^reference^	
	2^nd^ (1.02–1.08)	0.92 (0.78–1.09)	0.341	0.91 (0.73–1.14)	0.416
	3^rd^ (≥1.09)	1.09 (0.92–1.30)	0.321	1.20 (0.96–1.49)	0.106
Model 4	1^st^ (≤1.01)	1^reference^		1^reference^	
	2^nd^ (1.02–1.08)	0.89 (0.75–1.05)	0.170	0.87 (0.69–1.08)	0.204
	3^rd^ (≥1.09)	0.95 (0.80-1-14)	0.607	1.01 (0.81–1.27)	0.919
***Participants with coronary artery disease (n = 2407)***					
Model 1	1^st^ (≤1.01)	1^reference^		1^reference^	
	2^nd^ (1.02–1.07)	0.94 (0.79–1.13)	0.528	0.95 (0.75–1.19)	0.631
	3^rd^ (≥1.08)	1.30 (1.09–1.55)	0.003	1.43 (1.15–1.78)	0.001
Model 2	1^st^ (≤1.01)	1^reference^		1^reference^	
	2^nd^ (1.02–1.07)	0.90 (0.75–1.07)	0.231	0.89 (0.71–1.12)	0.324
	3^rd^ (≥1.08)	1.24 (1.03–1.48)	0.020	1.35 (1.08–1.69)	0.009
***Participants without coronary artery disease (n = 657)***					
Model 1	1^st^ (≤1.00)	1^reference^		1^reference^	
	2^nd^ (1.01–1.06)	1.30 (0.78–2.17)	0.318	2.01 (0.89–4.55)	0.095
	3^rd^ (≥1.07)	1.63 (1.00–2.65)	0.050	3.83 (1.81–8.10)	<0.001
Model 2	1^st^ (≤1.00)	1^reference^		1^reference^	
	2^nd^ (1.01–1.06)	1.30 (0.77–2.17)	0.326	1.85 (0.81–4.22)	0.142
	3^rd^ (≥1.07)	1.51 (0.92–2.49)	0.103	3.27 (1.53–7.00)	0.002

### Adding the INR to risk prediction models

We calculated a basic risk prediction model for the LURIC participants based on the cardiovascular risk factors age, sex, BMI, LDL-C- HDL-C, hypertension, smoking and diabetes mellitus. Adding the INR to this basic model improved the AUC significantly from 0.758 (0.740–0.776) to 0.764 (0.745–0.782) (**Table 4**). A similar increase in predictive performance could be observed when only the coronary artery disease patients were included. However, restricting the analysis to the patients with heart failure showed no additional benefit in risk prediction when adding the INR to the base model.

**Table 4:** Risk prediction models with and without inclusion of the INR.

	Harrells C	AUC (95% CI)	P*
*All participants (n = 3064)*			
Base^§^	0.719	0.758 (0.740–0.776)	
Base + INR	0.723	0.764 (0.745–0.782)	0.003
*Only CAD patients (n = 2407)*			
Base	0.701	0.741 (0.721–0.762)	
Base + INR	0.706	0.749 (0.728–0.769)	0.006
*Only heart failure patients (n = 916)*			
Base	0.657	0.710 (0.677–0.744)	
Base + INR	0.659	0.712 (0.679–0.745)	0.388

§ base model included age, sex, BMI, LDL-C- HDL-C, hypertension, smoking and diabetes mellitus

* Model including INR vs base model

Regarding reclassification, we only observed statistically significant improvements upon the addition of the INR to our basic risk model for the continuous NRI and the IDI, not for the predefined risk categories (Table 5). The improvements were modest and slightly higher in the CAD patients as compared to the whole cohort.

**Table 5:** Impact of INR on mortality: Net-reclassification-index and integrated-discrimination-index.

	All participants		Only CAD patients	
	Estimate	P	Estimate	P
NRI–categorical^§^*	0.0175 (-0.0143–0.0492)	0.280	0.0159 (-0.0412–0.0731)	0.585
NRI- continous^§^	0.088 (0.0122–0.1638)	0.023	0.1185 (0.0357–0.2012)	0.005
IDI^§^	0.0035 (0.0011–0.0059)	0.005	0.0045 (0.0015–0.0075)	0.003

§ base model including age, sex, BMI, LDL-C- HDL-C, hypertension, smoking and diabetes mellitus versus base model plus INR.

*Risk categories for categorical NRI were <5%, 5–10%, 10–20% and > 20%.

## Discussion

The main finding of our study is the significant direct association of the INR with all-cause and cardiovascular mortality in patients undergoing coronary angiography not receiving coumarin treatment which was most evident in patients with angiographic coronary artery disease after adjustment for conventional cardiovascular risk factors. However, adjusting the risk prediction models for NT-proBNP rendered the association insignificant.

Patients suffering from atherosclerosis and coronary heart disease are at increased risk of myocardial infarction or stroke due to the rupture of atherosclerotic plaques. At the first glance, this group of patients should derive benefit from a higher INR indicating lower thrombotic potential. Surprisingly, in our study we observe the contrary. An increase in the INR was associated with increased mortality. Examining the association of the INR with mortality along different INR values revealed a minimally increased mortality risk for INR values below 1.0, but a strongly increased risk for INR values above ca. 1.05. In patients free of CAD, the hazard ratio plots looked different with an increasing risk only up to ca. 1.1 but confidence intervals are quite large due to the smaller number of samples and events in this group.

Our results showing an apparently beneficial effect of higher blood clotting potential in CAD patients are in line with the recently published observations that there is an inverse association between the endogenous thrombin potential (ETP) and mortality in the LURIC study with the lowest hazard ratio in the 4th quartile of the ETP [14]. These observations may also reflect the so called “thrombin paradox” [15] that suggests an association of moderately elevated thrombin activation with a reduced cardiovascular risk [16]. Besides its well-established pro-coagulant functions (activation of platelets, fibrinogen, and other coagulation factors), thrombin, together with thrombomodulin, is also an activator of the key anti-coagulant protein C [17] which itself has direct cytoprotective effects that may slow the progression of CAD [17]. Our data are also in line with a recent publication that reported an inverse association of prothrombin time/INR with risk in patients with ACS [8] and we observe a similar association in our subgroup of patients with ACS (Figure D in S1 File).

Imaging studies have observed platelets inside carotid atherosclerotic plaques in the vicinity of leaky micro-vessels and intraplaque haemorrhages [18]. Plaque haemorrhage is more and more regarded as a marker for unstable high-risk plaques and was significantly more often found in carotid artery plaques of symptomatic patients with recurrent events than in asymptomatic patients [19]. Furthermore, intraplaque haemorrhage has been reported to be more common and lethal in older patients as compared to younger patients [20]. Enhanced coagulation capability might help to limit the amount of intraplaque haemorrhage formation and thereby lead to a stabilization of atherosclerotic plaques, which in turn would lead to fewer acute cardiovascular events and in the end to decreased cardiovascular mortality.

Regarding the increased INR that has been reported in patients with acute decompensated heart failure [5] the authors suggested several explanations: (1) activation of the coagulation system resulting in the consumption of coagulation factors, (2) hepatic congestion caused by elevated right-sided pressure and (3) hemodilution. Our study population does not include patients with acute decompensated heart failure or acute liver disease, but we additionally adjusted our analyses for prevalent CAD, heart failure and liver function in terms of the MELD-XI score. Although the association of the INR with mortality was attenuated it remained significant.

However, adding NT-proBNP, a marker of heart failure and the best single mortality predictor in LURIC, further attenuated the association of the INR with mortality and rendered it insignificant. This suggests that the increase in the INR might be caused by gradually increasing heart failure reflected by rising NT-proBNP concentrations that then might lead to liver congestion and a reduced production of coagulation factors in the liver. In line with this reasoning we noticed a decline of the concentration of coagulation factors II, V and VII that are mainly synthesized by the liver with rising INR whereas there were direct associations of the INR with factor VIII and von Willebrand factor that are both synthesized in different cells and tissues, not exclusively in the liver.

Adding markers of liver function such as GGT, albumin or cholinesterase to the risk prediction models attenuated the association of the INR with mortality and rendered it insignificant (**Figure F in S1 File**). Therefore, we suggest subclinical heart failure resulting in liver damage and reduced concentrations of the liver-derived coagulation factors II, V and VII as a possible mechanism mediating the increase in INR.

Regarding the medication of our study participants (**Table A in S1 File**) we observed a higher percentage of patients taking diuretics, ACE inhibitors and digitalis in the higher INR tertiles. This also reflects the higher percentage of patients suffering from heart failure. A higher overall drug intake may harm the liver by itself. However, additional adjustment for these drugs had only a minor effect on the association of the INR with mortality (data not shown).

### Limitations

All LURIC participants were of European origin and were recruited at a single tertiary referral centre. Therefore, our findings may not be representative for a random population sample or applicable to other ethnicities. Among the LURIC participants with CAD only 25.6% had a NT-proBNP below 125 ng/l suggesting they were free from heart failure. Furthermore, the INR and all biochemical markers were only measured once in baseline samples and values may vary over time.

## Conclusion

In the LURIC study, the INR was positively associated with all-cause and cardiovascular mortality in patients with prevalent CAD not on oral anticoagulant therapy as well as in patients without CAD, even after adjustment for prevalent heart failure and liver function. However, adjustment for NT-proBNP abolished the association suggesting clinical or subclinical heart failure leading to impaired liver function as being the underlying cause for increased INR and higher mortality.

## Supporting information

S1 File Supplementary tables and figures. (DOCX)
