# Supplementary material for: The association of high-normal international-normalized-ratio (INR) with mortality in patients referred for coronary angiography
Source: PLoS One. 2019 Aug 15;14(8):e0221112. doi: 10.1371/journal.pone.0221112 (PMC6695160; doi:10.1371/journal.pone.0221112)
Supplement: S1 File — (DOCX) [file pone.0221112.s001.docx]

**The association of high-normal international-normalized-ratio (INR) with mortality in patients referred for coronary angiography**

Graciela E. Delgado^1^, Andreas Zirlik^2^, Rudolf Gruber^3^, Thomas Scheffold^4^, Bernhard K. Krämer^1^, Winfried März^1,5,6^, Marcus E. Kleber^1^.

 ^1^ Vth Department of Medicine, Medical Faculty Mannheim, Heidelberg University, Germany;

^2^ University Heart Centre Freiburg University, Department of Cardiology and Angiology I, Faculty of Medicine, University of Freiburg, Germany

^3^ Hospital of the Order of St.John of God, Central Laboratory, Regensburg, Germany;

^4^ MediClin Medical Care Centre, Department of Cardiology, Lahr, Germany

^5^ Clinical Institute of Medical and Chemical Laboratory Diagnostics, Medical University
Graz, Graz, Austria;

^6^ Synlab Academy, Synlab Holding Deutschland GmbH, Mannheim, Germany

Content

[Supplementary Tables 3](#_Toc15739697)

[Table A. Medication of study participants according to INR tertiles 3](#_Toc15739698)

[Table B. Quick tertiles and mortality 4](#_Toc15739699)

[Table C. aPTT tertiles and mortality 5](#_Toc15739700)

[Table D. Patient characteristics according to heart failure status 6](#_Toc15739701)

[Supplementary Figures 7](#_Toc15739702)

[Fig A. Histogram of the distribution of INR values in LURIC. 7](#_Toc15739703)

[Fig B. Association of INR and cardiovascular mortality, stratified for CAD status. 8](#_Toc15739704)

[Fig C. Association of INR and mortality, stratified for heart failure. 9](#_Toc15739705)

[Fig D. Association of INR and mortality, stratified for acute coronary syndrome. 10](#_Toc15739706)

[Fig E. Relative importance of variables included in Cox regression analysis. 11](#_Toc15739707)

[Fig F. Association of INR and mortality, additionally adjusted for markers of liver function. 12](#_Toc15739708)

# Supplementary Tables

## Table A. Medication of study participants according to INR tertiles

|  | INR | | |  |
| --- | --- | --- | --- | --- |
|  | 1st tertile | 2nd tertile | 3rd tertile | P |
| Antibiotics (%) | 1.3 | 1.1 | 2.6 | 0.025 |
| Antiplatelet drugs (%) | 77.1 | 77.9 | 71.5 | 0.002 |
| ACE inhibitor (%) | 47.5 | 54.6 | 57.7 | <0.001 |
| AT2 receptor blocker (%) | 4.6 | 4.2 | 3.8 | 0.689 |
| Beta blocker (%) | 64.8 | 65.3 | 62.4 | 0.386 |
| Calcium antagonist (%) | 13.7 | 15.8 | 15.5 | 0.315 |
| Digitalis (%) | 8.1 | 11.2 | 20.4 | <0.001 |
| Diuretics (%) | 22.4 | 26.2 | 33.7 | <0.001 |
| Glucocorticoids (%) | 2.3 | 1.9 | 2.1 | 0.799 |
| GP IIb/IIIa blocker (%) | 0.3 | 0.2 | 0.1 | 0.741 |
| Hormonal replacement therapy (%) | 5.2 | 3.8 | 2.7 | 0.020 |
| Insulin (%) | 4.5 | 5.7 | 6.0 | 0.291 |
| Nitrates (%) | 31.2 | 33.4 | 35.5 | 0.119 |
| Statins (%) | 48.7 | 47.3 | 46.8 | 0.690 |
| Oral antidiabetic drug (%) | 7.0 | 8.1 | 10.7 | 0.010 |
| Theophyllin/bronchodilator (%) | 7.1 | 5.1 | 6.3 | 0.156 |
| Uric acid lowering medication (%) | 0.3 | 0.5 | 0.9 | 0.138 |
| Vitamin K antagonist (%) | 0.0 | 0.0 | 0.0 | - |

## Table B. Quick tertiles and mortality

|  |  | **All-cause mortality** |  | **Cardiovascular mortality** |  |
| --- | --- | --- | --- | --- | --- |
|  |  | HR (95% CI) | *p* | HR (95% CI) | *P* |
| ***All participants*** |  |  |  |  |  |
| Model 1 | 1^st^ (≤32) | 1^reference^ |  | 1^reference^ |  |
|  | 2^nd^ (33-35) | 1.02 (0.86-1.21) | 0.800 | 1.04 (0.83-1.29) | 0.746 |
|  | 3^rd^ (≥36) | 1.34 (1.14-1.58) | <0.001 | 1.56 (0.97-1.44) | 0.091 |
|  |  |  |  |  |  |
| Model 2 | 1^st^ (≤32) | 1^reference^ |  | 1^reference^ |  |
|  | 2^nd^ (33-35) | 0.96 (0.81-1.14) | 0.678 | 0.96 (0.78-1.19) | 0.714 |
|  | 3^rd^ (≥36) | 1.26 (1.07-1.50) | 0.006 | 1.15 (0.95-1.41) | 0.159 |
|  |  |  |  |  |  |
| ***Participants with coronary artery disease*** | | |  |  |  |
| Model 1 | 1^st^ (≤32) | 1^reference^ |  | 1^reference^ |  |
|  | 2^nd^ (33-35) | 1.02 (0.85-1.21) | 0.858 | 0.94 (0.75-1.17) | 0.571 |
|  | 3^rd^ (≥36) | 1.24 (1.06-1.47) | 0.009 | 1.16 (0.95-1.43) | 0.152 |
|  |  |  |  |  |  |
| Model 2 | 1^st^ (≤32) | 1^reference^ |  | 1^reference^ |  |
|  | 2^nd^ (33-35) | 1.00 (0.84-1.07) | 0.982 | 0.94 (0.75-1.17) | 0.558 |
|  | 3^rd^ (≥36) | 1.18 (1.00-1.40) | 0.054 | 1.14 (0.92-1.40) | 0.236 |
|  |  |  |  |  |  |
| ***Participants without coronary artery disease*** | | |  |  |  |
| Model 1 | 1^st^ (≤31) | 1^reference^ |  | 1^reference^ |  |
|  | 2^nd^ (32-34) | 1.69 (1.03-2.75) | 0.036 | 2.55 (1.31-4.99) | 0.006 |
|  | 3^rd^ (≥35) | 1.53 (0.93-2.54) | 0.096 | 1.81 (0.88-3.72) | 0.108 |
|  |  |  |  |  |  |
| Model 2 | 1^st^ (≤31) | 1^reference^ |  | 1^reference^ |  |
|  | 2^nd^ (32-34) | 1.71 (1.04-2.80) | 0.033 | 2.58 (1.31-5.07) | 0.006 |
|  | 3^rd^ (≥35) | 1.55 (0.93-2.58) | 0.093 | 1.82 (0.88-3.79) | 0.108 |
|  |  |  |  |  |  |

## Table C. aPTT tertiles and mortality

|  |  | **All-cause mortality** |  | **Cardiovascular mortality** |  |
| --- | --- | --- | --- | --- | --- |
|  |  | HR (95% CI) | *p* | HR (95% CI) | *P* |
| ***All participants*** |  |  |  |  |  |
| Model 1 | 1^st^ (≤32) | 1^reference^ |  | 1^reference^ |  |
|  | 2^nd^ (33-35) | 1.04 (0.88-1.23) | 0.648 | 0.97 (0.79-1.20) | 0.778 |
|  | 3^rd^ (≥36) | 1.26 (1.08-1.47) | 0.003 | 1.19 (0.97-1.44) | 0.091 |
|  |  |  |  |  |  |
| Model 2 | 1^st^ (≤32) | 1^reference^ |  | 1^reference^ |  |
|  | 2^nd^ (33-35) | 1.02 (0.86-1.20) | 0.848 | 0.96 (0.78-1.19) | 0.714 |
|  | 3^rd^ (≥36) | 1.20 (1.02-1.40) | 0.024 | 1.15 (0.95-1.41) | 0.159 |
|  |  |  |  |  |  |
| ***Participants with coronary artery disease*** | | |  |  |  |
| Model 1 | 1^st^ (≤32) | 1^reference^ |  | 1^reference^ |  |
|  | 2^nd^ (33-35) | 1.02 (0.85-1.21) | 0.858 | 0.94 (0.75-1.17) | 0.571 |
|  | 3^rd^ (≥36) | 1.24 (1.06-1.47) | 0.009 | 1.16 (0.95-1.43) | 0.152 |
|  |  |  |  |  |  |
| Model 2 | 1^st^ (≤32) | 1^reference^ |  | 1^reference^ |  |
|  | 2^nd^ (33-35) | 1.00 (0.84-1.07) | 0.982 | 0.94 (0.75-1.17) | 0.558 |
|  | 3^rd^ (≥36) | 1.18 (1.00-1.40) | 0.054 | 1.14 (0.92-1.40) | 0.236 |
|  |  |  |  |  |  |
| ***Participants without coronary artery disease*** | | |  |  |  |
| Model 1 | 1^st^ (≤31) | 1^reference^ |  | 1^reference^ |  |
|  | 2^nd^ (32-34) | 1.69 (1.03-2.75) | 0.036 | 2.55 (1.31-4.99) | 0.006 |
|  | 3^rd^ (≥35) | 1.53 (0.93-2.54) | 0.096 | 1.81 (0.88-3.72) | 0.108 |
|  |  |  |  |  |  |
| Model 2 | 1^st^ (≤31) | 1^reference^ |  | 1^reference^ |  |
|  | 2^nd^ (32-34) | 1.71 (1.04-2.80) | 0.033 | 2.58 (1.31-5.07) | 0.006 |
|  | 3^rd^ (≥35) | 1.55 (0.93-2.58) | 0.093 | 1.82 (0.88-3.79) | 0.108 |
|  |  |  |  |  |  |

## Table D. Patient characteristics according to heart failure status

| Variable | No HF  (N=2148) | HFrEF  (N=505) | HFpEF  (N=411) | P |
| --- | --- | --- | --- | --- |
| Age (years) | 61.3(10.8) | 64.1(10.4) | 66.6(9.3) | <0.001 |
| Male sex (%) | 68.7 | 78.4 | 62.8 | <0.001 |
| hsCRP (mg/l) | 2.92(1.18-7.39) | 5.8(1.96-11.5) | 3.91(1.52-9.36) | <0.001 |
| NT-proBNP (ng/ml) | 179(82-447) | 1297(504-2883) | 497(231-1216) | <0.001 |
| TnT (pg/ml) | 8.0(4.0-18.4) | 27.0(13.0-63.0) | 12.0(6.89-23.2) | <0.001 |
| Albumin (g/dl) | 4.42(0.54) | 4.26(0.55) | 4.35(0.57) | <0.001 |
| Cholinesterase (U/l) | 5822(1277) | 5371(1465) | 5662(1272) | <0.001 |
| GOT (U/l) | 11.6(7.81) | 12.2(7.51) | 11.8(7.44) | 0.288 |
| Bilirubin (mg/dl) | 0.62(0.36) | 0.66(0.39) | 0.64(0.37) | 0.038 |
| MELD-XI | 5.6(3.3-7.6) | 6.4(4.2-9.4) | 5.8(3.3-8.2) | <0.001 |
| FII (U/dl) | 110(18.7) | 106(22.1) | 104(22.3) | <0.001 |
| FV (U/dl) | 115(21.0) | 111(22.8) | 113.6(20.1) | 0.010 |
| FVII (U/dl) | 127(25.4) | 119(30.9) | 123(24.7) | <0.001 |
| FVIII (U/dl) | 169(66.1) | 203(78.4) | 179(65.7) | <0.001 |
| von Willebrandt (U/dl) | 159(65.44) | 199(83.42) | 174(76.84) | <0.001 |
| tPA/PAI-1 complex (µg/l) | 7.64(3.81) | 9.10(4.17) | 8.64(4.01) | <0.001 |
| tPA activity (U/l) | 0.63(0.34-0.98) | 0.68(0.32-1.15) | 0.61(0.32-0.98) | 0.162 |
| PAI-1 activity (U/ml) | 18(10-33) | 17(10-35) | 22(11-38) | 0.206 |
| D-Dimer (mg/l) | 0.34(0.22-0.58) | 0.50(0.25-0.99) | 0.37(0.23-0.67) | <0.001 |
| Fibrinogen (mg/dl) | 386(102.7) | 437(120.6) | 396(103.3) | <0.001 |
| aPTT (sec) | 33(31-36) | 33(31-37) | 33(31-36) | 0.021 |
| INR | 1.04(0.08) | 1.08(0.11) | 1.07(0.11) | <0.001 |
| ETP (nmol*min) | 100(23.1) | 96.7(26.8) | 94.6(24.9) | <0.001 |
| Platelets (/nl) | 235(64.7) | 234(77.6) | 231(71.4) | 0.645 |
| eGFR (ml/min/1.73 m2) | 85.4(18.9) | 74.3(21.4) | 76.4(19.2) | <0.001 |

*ANOVA for continuous variables (non-normally distributed variables were log transformed before entering analyses), χ^2^ test for categorical variables. HF: heart failure; HFrEF: heart failure with reduced ejection fraction; HFpEF: heart failure with preserved ejection fraction.

# Supplementary Figures


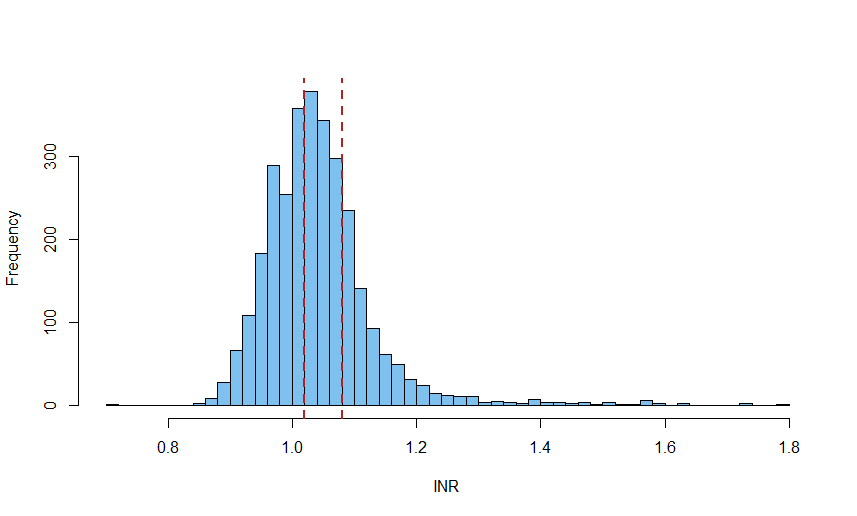


Fig A. Histogram of the distribution of INR values in LURIC. Tertile boundaries are shown as dashed red lines.


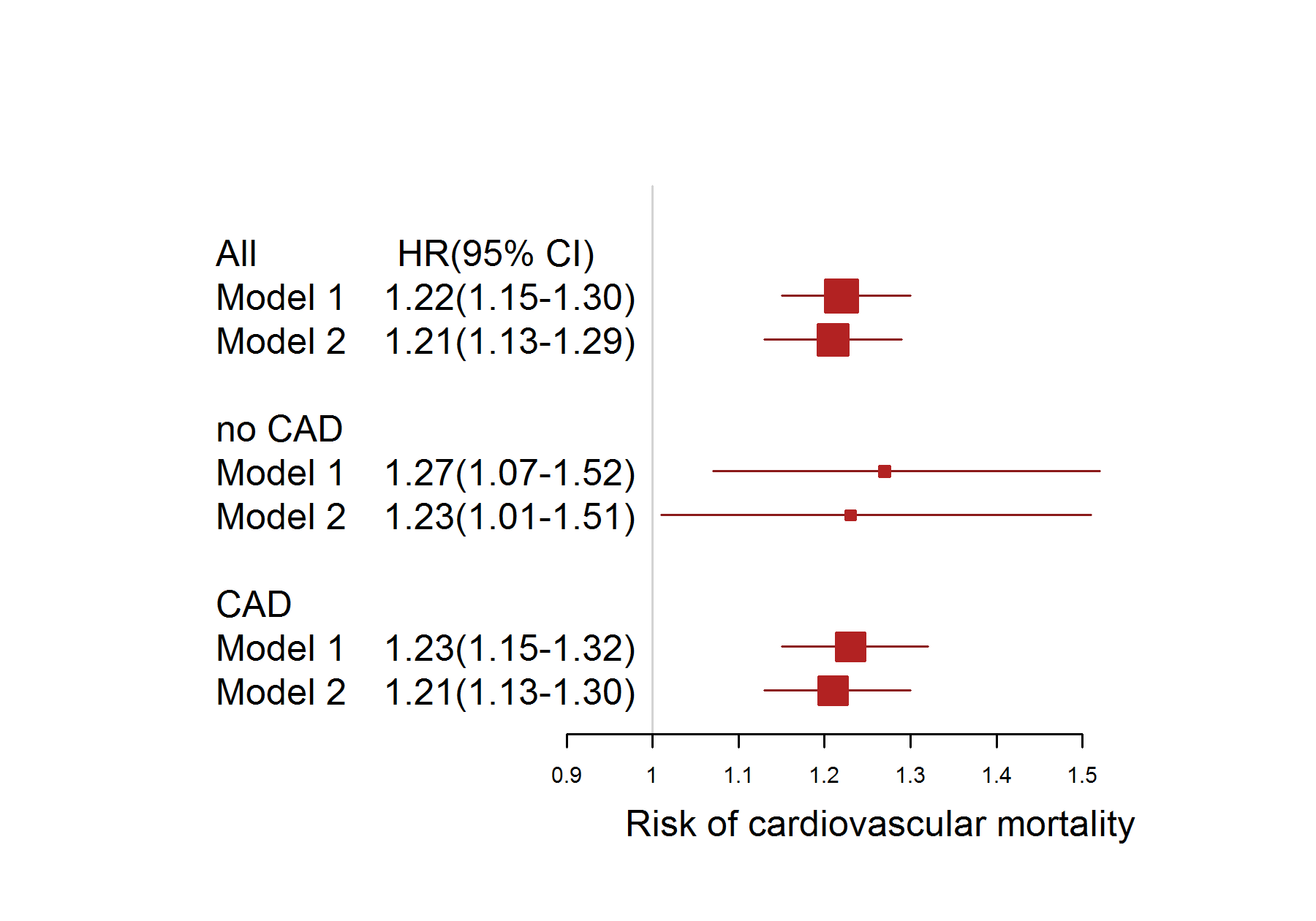


Fig B. Association of INR and cardiovascular mortality, stratified for CAD status. Cox proportional hazard regression has been used to calculate the risk of cardiovascular mortality per 1-SD increase in INR stratified for CAD status. Model 1: adjusted for age and gender; model 2: additionally adjusted for BMI, LDL-C, HDL-C, hypertension, diabetes mellitus, smoking status and the use of antiplatelet and lipid lowering drugs.


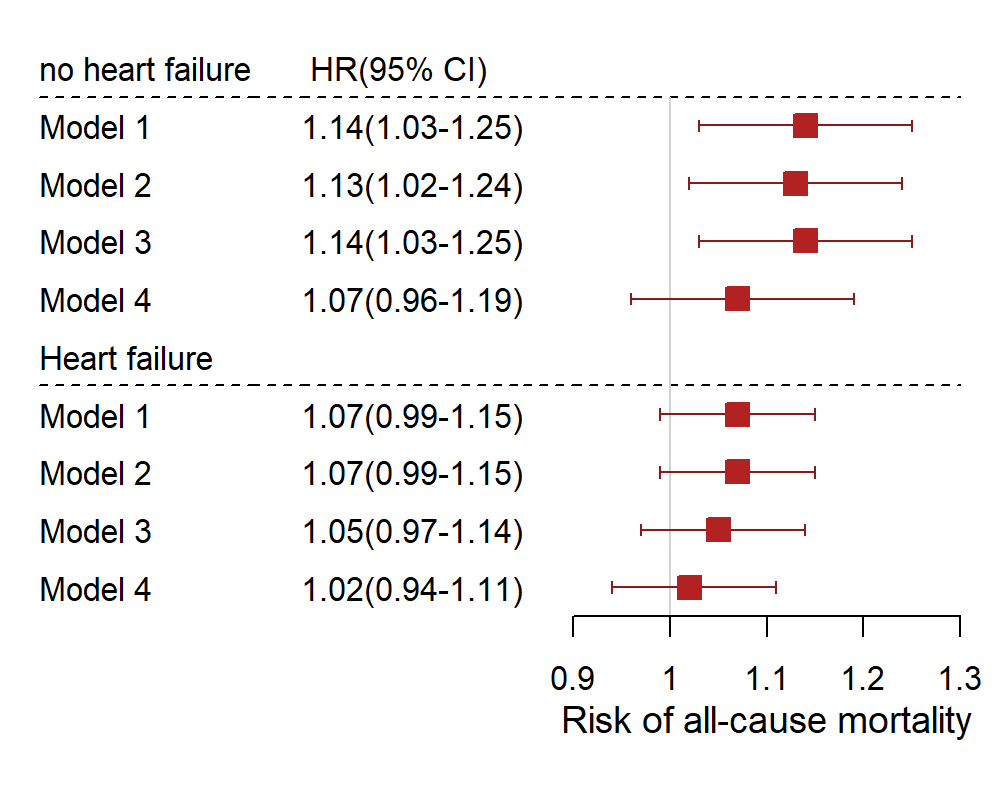


Fig C. Association of INR and mortality, stratified for heart failure. Cox proportional hazard regression has been used to calculate the risk of all-cause mortality per 1-SD increase in INR stratified for heart failure. Model 1: adjusted for age and gender; model 2: additionally adjusted for BMI, LDL-C, HDL-C, hypertension, diabetes mellitus, smoking status and the use of antiplatelet and lipid lowering drugs; model 3: additionally adjusted for MELD-XI score and history of CAD; model 4: model 3 + NT-proBNP.


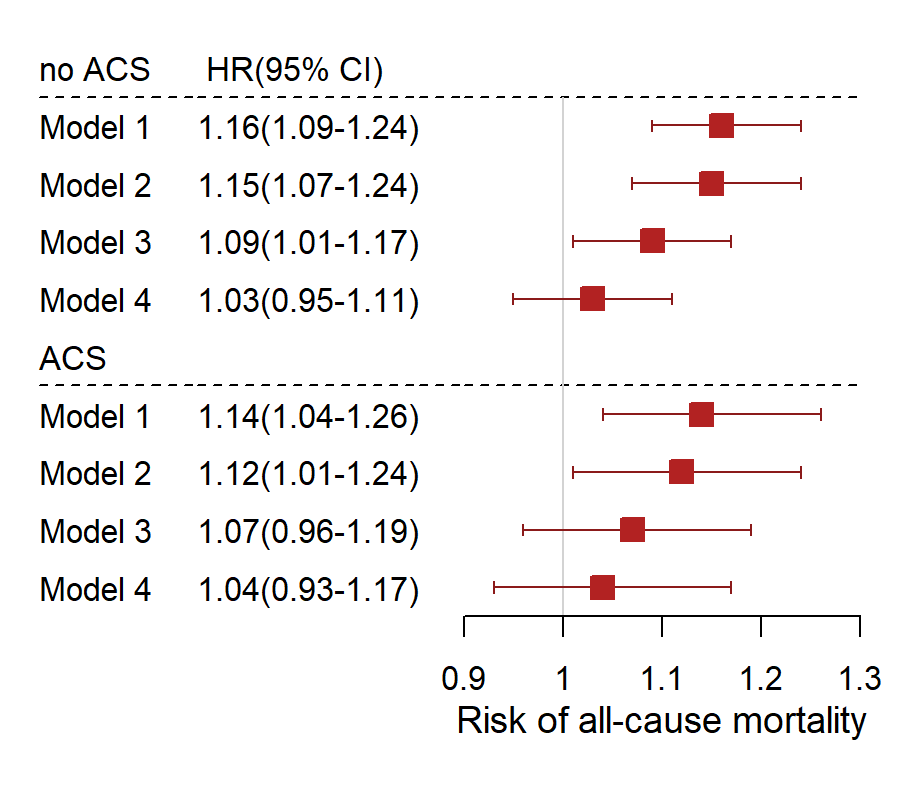


Fig D. Association of INR and mortality, stratified for acute coronary syndrome. Cox proportional hazard regression has been used to calculate the risk of all-cause mortality per 1-SD increase in INR stratified for acute coronary syndrome (ACS). Model 1: adjusted for age and gender; model 2: additionally adjusted for BMI, LDL-C, HDL-C, hypertension, diabetes mellitus, smoking status and the use of antiplatelet and lipid lowering drugs; model 3: additionally adjusted for MELD-XI score and history of CAD or heart failure; model 4: model 3 + NT-proBNP.


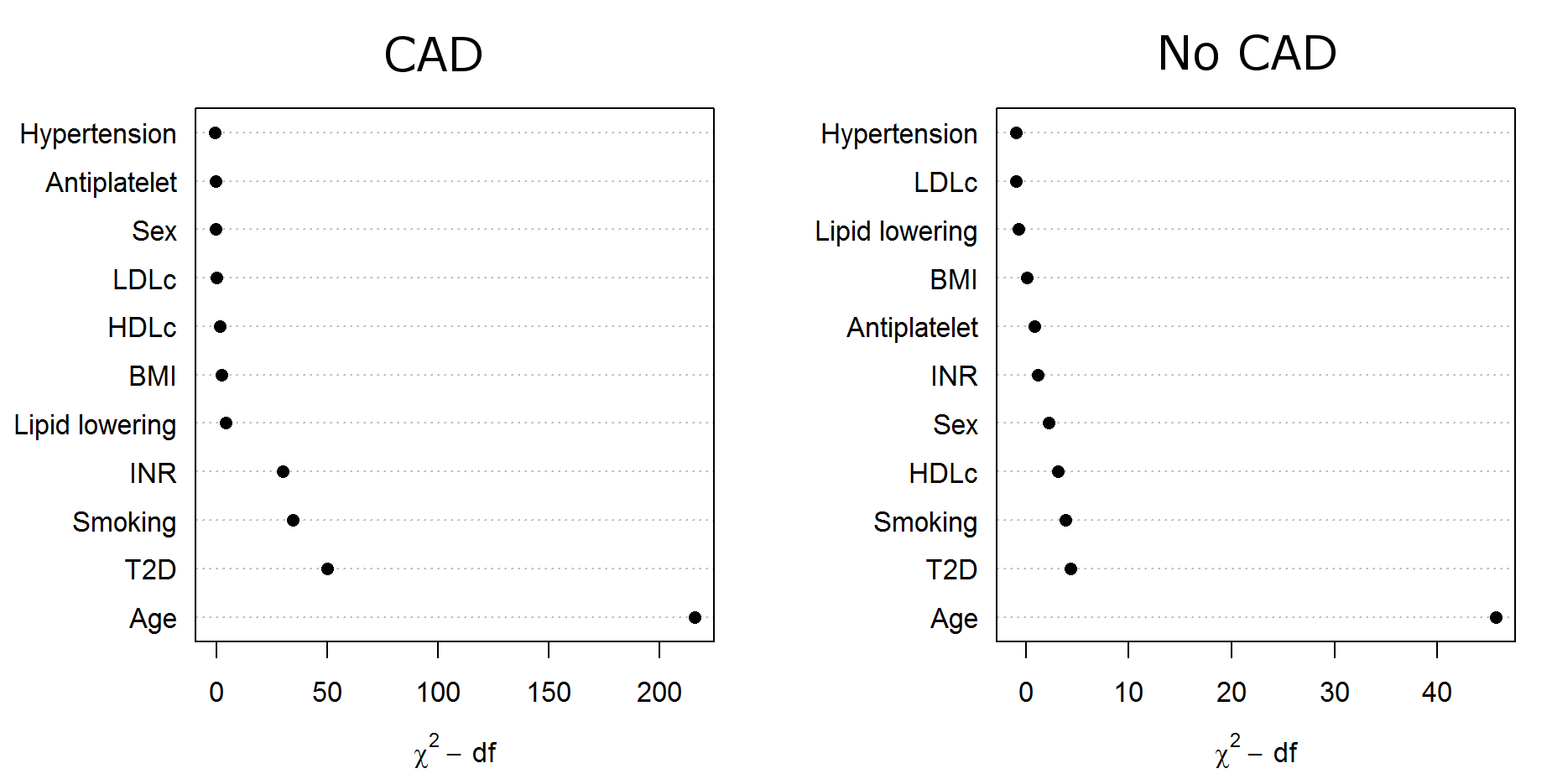


Fig E. Relative importance of variables included in Cox regression analysis. The relative importance of variables entered in Cox regression analysis of all-cause mortality in terms of χ^2^ – degrees of freedom. Antiplatelet: antiplatelet therapy; HDLc: HDL-cholesterol; INR: international-normalized-ratio; LDLc: LDL-cholesterol; Lipid lowering: lipid lowering therapy; T2D: type 2 diabetes


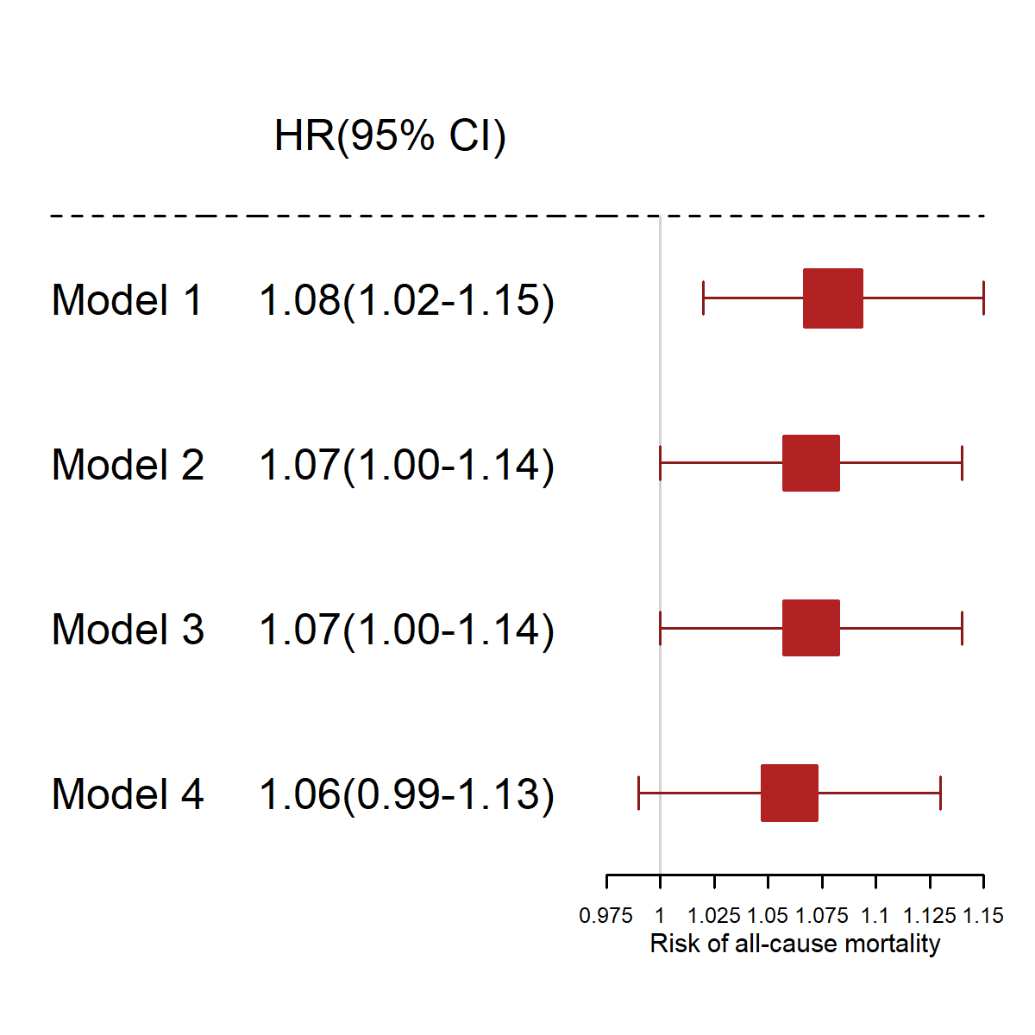


Fig F. Association of INR and mortality, additionally adjusted for markers of liver function. Cox proportional hazard regression has been used to calculate the risk of all-cause mortality per 1-SD increase in INR. Model 1: adjusted for age, gender, BMI, LDL-C, HDL-C, hypertension, diabetes mellitus, smoking status and the use of antiplatelet and lipid lowering drugs, MELD-XI score and history of CAD or heart failure; model 2: model 1 + GGT; model 3: model 1 + albumin; model 4: model 1 + cholinesterase.
